# Nutritional Imbalance and Oral Functional Limitation Jointly Associated with Depressive Symptoms in Adults Living Alone: A Nationally Representative Cross-Sectional Study

**DOI:** 10.3390/nu18132055

**Published:** 2026-06-24

**Authors:** Young-Suk Jung, Hyunjoo Joo

**Affiliations:** 1 Department of Periodontology, School of Dentistry, Chonnam National University, Gwangju 61186, Republic of Korea; noblesoul@hanmail.net; 2 Leader’s Dental Clinic, Jeju 63560, Republic of Korea; 3 Department of Preventive Medicine, College of Medicine, Dankook University, Cheonan 31116, Republic of Korea

**Keywords:** nutritional imbalance, oral functional limitation, depressive symptoms, adults living alone, metabolic syndrome, KNHANES, mental health, public health nutrition

## Abstract

**Background/Objectives:** Adults living alone are vulnerable to nutritional inadequacy and depressive symptoms; however, their combined influence remains poorly understood. This study examined the independent and joint associations of nutritional imbalance and oral functional limitation with depressive symptoms among adults living alone and explored potential indirect pathways involving metabolic syndrome (exploratory). **Methods:** This cross-sectional study used nationally representative data from the Korea National Health and Nutrition Examination Survey (KNHANES) 2022 and 2024, including 1572 adults aged ≥19 years living alone. Nutritional imbalance was assessed by the number of essential nutrients consumed below the Estimated Average Requirement (EAR; range 0–8). Oral functional limitation was defined as chewing or speaking difficulty. Depressive symptoms were evaluated using the Patient Health Questionnaire-9 (PHQ-9). Survey-weighted regression and bootstrapped indirect-effect analyses were performed. **Results:** Nutritional imbalance was associated with higher PHQ-9 scores (β = 0.216; 95% CI: 0.098–0.335). Oral functional limitation (β = 1.278; 95% CI: 0.739–1.816) and metabolic syndrome score (β = 0.156; 95% CI: 0.006–0.306) were independently associated with depressive severity. Participants with both high nutritional imbalance and oral functional limitation had substantially higher odds of clinical depression (PHQ-9 ≥ 10; OR = 4.268; 95% CI: 2.037–8.943) than the reference group. Exploratory analyses suggested that indirect effects via oral functional limitation and metabolic syndrome were directionally consistent but did not reach statistical significance. **Conclusions:** Nutritional imbalance and oral functional limitation were jointly associated with depressive symptoms among adults living alone. These findings highlight the importance of integrated strategies targeting dietary quality, oral health, and metabolic health in vulnerable single-person households.

## 1. Introduction

The proportion of single-person households has increased substantially in Korea, rising from 15.5% of all households in 2000 to approximately 35% in 2023, driven by urbanization, delayed marriage, population aging, and rising divorce rates [1]. Adults living alone face a distinct set of health disadvantages compared with those living with others, including reduced meal regularity, lower dietary diversity, and greater reliance on convenience or processed foods [2,3]. These nutritional vulnerabilities are compounded by social isolation, which itself is an independent risk factor for depressive symptoms [4].

Depression is among the most burdensome non-communicable conditions globally and is disproportionately prevalent among socially isolated adults [5]. In Korea, single-person households have been found to report higher rates of depressive symptoms than multi-person households, even after adjustment for sociodemographic characteristics [6,7]. However, the pathways through which living alone translates into greater depressive symptom burden remain incompletely characterized. Nutritional imbalance has been proposed as one plausible mechanism, given established links between micronutrient deficiency and neurobiological processes relevant to mood regulation, including serotonin synthesis, hypothalamic–pituitary–adrenal axis function, and systemic inflammation [8,9].

Oral functional limitation—encompassing chewing difficulty and speaking difficulty—may represent a clinically relevant factor related to depressive symptoms in nutritionally vulnerable individuals. Impaired masticatory function has been associated with reduced intake of protein, calcium, and other key nutrients [10,11]. Prior work using Korean national survey data has demonstrated that chewing difficulty is independently associated with depressive symptoms, partly through health-related quality of life [12,13,14]. Whether oral functional limitation is associated with the relationship between nutritional imbalance and depressive symptoms in single-person households has not been examined.

Metabolic syndrome, characterized by clustering of abdominal obesity, dyslipidemia, hypertension, and impaired fasting glucose, has also been linked to depressive symptoms through inflammatory and neurobiological mechanisms [15]. Single-person households show elevated prevalence of metabolic syndrome relative to multi-person households [16]. However, whether metabolic syndrome may operate as a potential intermediate in the nutritional imbalance–depression relationship in this population has not been investigated.

Despite growing evidence linking both nutritional imbalance and oral functional limitation to mental health outcomes, their combined influence on depressive symptoms among adults living alone has not been systematically examined. Using nationally representative KNHANES 2022 and 2024 data, this study examined the independent and joint associations of nutritional imbalance and oral functional limitation with depressive symptom burden among adults living alone, while additionally exploring potential indirect pathways involving metabolic syndrome (exploratory). To our knowledge, this is the first study to investigate these joint nutritional and oral health patterns in relation to depressive symptoms among adults living alone, who face a heightened risk of nutrient inadequacy.

## 2. Materials and Methods

### 2.1. Study Design and Data Source

This cross-sectional study used data from KNHANES, an annual nationally representative survey of the non-institutionalized Korean population conducted by the Korea Disease Control and Prevention Agency (KDCA). KNHANES employs a stratified, multistage probability sampling design and collects data through health interviews, physical examinations, and nutrition surveys [17]. Data from the 2022 and 2024 survey cycles were used; the 2023 cycle was excluded because PHQ-9 data were not collected in that year. Data from the 2022 and 2024 survey cycles were pooled to increase the analytic sample size for this subpopulation analysis. KNHANES is conducted as an independent annual cross-sectional survey with a new probability sample drawn each year, and participants are not followed across cycles; the pooled dataset therefore constitutes a single cross-sectional sample. Pooling multiple independent KNHANES cycles is a standard approach in Korean nutritional epidemiology and public health research. When pooling the two cycles, nutritional survey weights were rescaled by dividing each cycle’s weight by the number of pooled cycles (i.e., multiplying by 0.5), following KDCA recommendations for multi-cycle KNHANES analyses. All KNHANES protocols were approved by the KDCA Institutional Review Board, and all participants provided written informed consent. This study involved secondary analysis of publicly available de-identified data and was exempt from additional ethical review.

### 2.2. Study Participants

**Figure 1:**
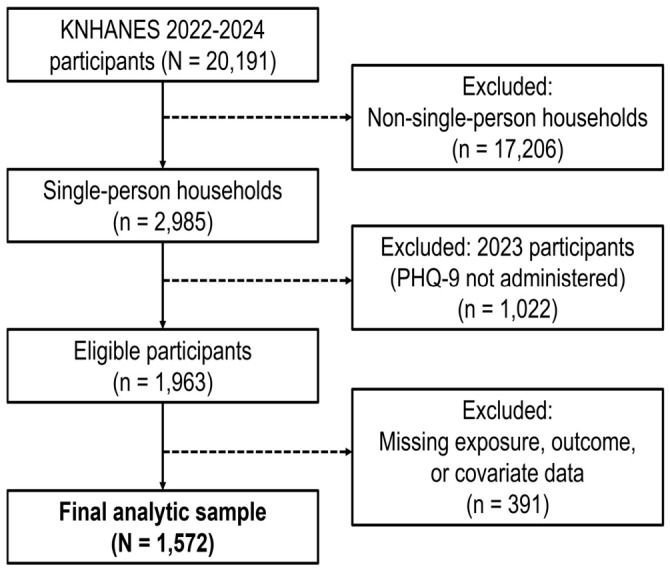
Flowchart of participant selection from KNHANES 2022–2024.

Among 20,191 participants in KNHANES 2022–2024, individuals aged < 19 years (*n* = 2929) and those residing in multi-person households (*n* = 14,277) were excluded. The 2023 cycle was excluded owing to the absence of PHQ-9 data (*n* = 1022). From the remaining 1963 adults in single-person households, participants with missing data on nutritional variables (*n* = 92), oral function (*n* = 73), metabolic or PHQ-9 variables, or any covariate (*n* = 226) were excluded, yielding a final analytic sample of 1572 participants (Figure 1).

### 2.3. Nutritional Imbalance

Dietary intake was assessed by a single 24 h dietary recall administered by trained interviewers. Nutritional imbalance was operationalized as the count of eight nutrients with intake below the sex- and age-specific estimated average requirement (EAR) according to the 2020 Korean Dietary Reference Intakes: protein, calcium, iron, vitamin A, vitamin B1, vitamin B2, niacin, and vitamin C. Scores ranged from 0 to 8, with higher scores indicating greater nutritional imbalance. Total energy intake was included as a continuous covariate rather than as a component of the imbalance index to avoid conflating energy sufficiency with nutrient quality. In the present study, the term ‘nutritional imbalance’ is used operationally to denote inadequate intake of essential nutrients, defined as intake below the sex- and age-specific EAR; nutrient excess was not assessed. These eight nutrients were selected because sex- and age-specific EAR values are defined for each in the 2020 KDRIs and because reliable adequacy assessment is feasible using the KNHANES 24 h recall instrument. Vitamin D was not included because dietary assessment from food sources alone substantially underestimates total vitamin D exposure, precluding reliable EAR-based classification in this dataset. Macronutrient balance (e.g., fat intake) was considered a distinct construct and was outside the scope of the present operational definition.

### 2.4. Oral Functional Limitation

Oral functional limitation was defined as the presence of chewing difficulty or speaking difficulty. Chewing difficulty was assessed using the KNHANES oral health questionnaire item on chewing problems (BM7), with response options of very uncomfortable, uncomfortable, fair, not uncomfortable, and not at all uncomfortable; participants reporting ‘very uncomfortable’ or ‘uncomfortable’ were classified as having chewing difficulty. Speaking difficulty was assessed using the corresponding item on speaking problems (BM8), using the same five-point response scale; participants reporting ‘very uncomfortable’ or ‘uncomfortable’ were classified as having speaking difficulty. Oral functional limitation was defined as the presence of either chewing difficulty or speaking difficulty.

### 2.5. Metabolic Syndrome

Metabolic syndrome burden was assessed using the count of NCEP-ATP III components met, with Korean abdominal obesity thresholds applied. Participants were classified as having metabolic syndrome if three or more of the following five criteria were met: (1) abdominal obesity (waist circumference ≥ 90 cm in men, ≥85 cm in women); (2) elevated triglycerides (≥150 mg/dL or lipid-lowering medication); (3) reduced HDL cholesterol (<40 mg/dL in men, <50 mg/dL in women, or lipid-lowering medication); (4) elevated blood pressure (systolic ≥ 130 mmHg or diastolic ≥85 mmHg or antihypertensive medication); (5) elevated fasting glucose (≥100 mg/dL or antidiabetic medication). For exploratory indirect-effect analyses, the number of metabolic syndrome components met (range 0–5) was used as a continuous variable to maximize statistical power.

### 2.6. Depressive Symptoms

Depressive symptom severity was assessed using the Korean version of the Patient Health Questionnaire-9 (PHQ-9), a validated nine-item self-report instrument with total scores ranging from 0 to 27 [18]. Higher scores indicate greater severity. The PHQ-9 continuous score was the primary outcome. For secondary analyses, clinically significant depressive symptoms were defined as PHQ-9 ≥ 10.

### 2.7. Covariates

Covariates included age (continuous), sex, education level (elementary or lower, middle school, high school, college or higher), household income (quintiles), marital status (married or partnered vs. single, separated, divorced, or widowed), employment status (employed vs. not employed), smoking status (current smoker vs. non- or former smoker), alcohol consumption (non-drinker, infrequent drinker, drinker), aerobic physical activity (meeting vs. not meeting guidelines), and total energy intake (continuous, kcal/day). Marital status was included as a covariate reflecting relationship and social support context, which may independently influence mental health outcomes irrespective of household composition. In Korea, single-person households include a substantial proportion of individuals who are married or partnered but reside alone (e.g., due to occupational relocation), and adjustment for marital status was therefore considered necessary to isolate the effect of nutritional and oral health factors.

### 2.8. Statistical Analysis

All analyses incorporated sampling weights, stratification variables, and cluster identifiers to account for the complex survey design of KNHANES, using the nutritional survey weight as the primary analytic weight. Continuous variables are reported as weighted mean (SD) and categorical variables as unweighted frequency (weighted percentage). Between-group differences were assessed using survey-weighted *t*-tests for continuous variables and Rao-Scott chi-square tests for categorical variables.

Indirect-effect analysis followed a product-of-coefficients approach using survey-weighted generalized linear models. The association between nutritional imbalance and oral functional limitation was estimated using logistic regression (yielding odds ratios); associations with PHQ-9 score and metabolic syndrome score were estimated using linear regression (yielding β coefficients). All models were mutually adjusted for the full set of covariates listed above. Indirect effects were estimated as the product of the relevant path coefficients; 95% confidence intervals were derived using 1000 bootstrap iterations with preservation of the stratified cluster structure of the complex survey design.

Nutritional imbalance was dichotomized at the sample median (score ≥ 5 = high imbalance) to facilitate joint exposure classification and to produce clinically interpretable risk estimates. The median was selected as the cut-point in the absence of an established clinical threshold for this composite index; sensitivity analyses using tertile-based classification yielded consistent findings (Supplementary Table S5).

Joint exposure analyses combined nutritional imbalance (dichotomized at the median, score ≥ 5 = high) and oral functional limitation (yes/no) into four mutually exclusive groups: neither condition, high nutritional imbalance only, oral functional limitation only, and both conditions. The group with neither condition served as the reference. Survey-weighted linear and logistic regression models were used to estimate associations with PHQ-9 score and PHQ-9 ≥ 10, respectively.

Sensitivity analyses used an alternative PHQ-9 cut-off of ≥5 for the binary outcome definition. Stratified analyses by sex and age group (<65 vs. ≥65 years) were conducted for the joint exposure outcome. Additive interaction between high nutritional imbalance and oral functional limitation was assessed using the relative excess risk due to interaction (RERI), attributable proportion (AP), and synergy index (SI), derived from the joint exposure logistic regression model. The 95% confidence interval for RERI was estimated using the delta method.

All analyses were performed using R version 4.5.2 (R Foundation for Statistical Computing, Vienna, Austria). Statistical significance was defined as a two-sided *p* < 0.05. This study was reported in accordance with the STROBE guidelines. PHQ-9 scores were right-skewed; residual diagnostics, including residual plots and Q-Q plots, were examined and indicated an acceptable fit for survey-weighted linear regression.

## 3. Results

### 3.1. Sample Characteristics

Figure 1 presents the participant selection process for the study population. Among adults participating in the Korea National Health and Nutrition Examination Survey (KNHANES) 2022 and 2024, individuals living in single-person households with complete information on depressive symptoms, nutritional intake, oral functional limitation, metabolic syndrome, and covariates were included in the final analysis. The final analytic sample comprised 1572 adults living alone, of whom 104 (6.6% weighted) had PHQ-9 scores ≥ 10.

The weighted mean age was 50.5 years (SD 19.4), and 49.0% were women. Compared with those without depressive symptoms, participants with PHQ-9 scores ≥ 10 were more likely to belong to the lowest income quintile (62.8% vs. 35.3%), to be current smokers (35.7% vs. 22.8%), and to report oral functional limitation (38.3% vs. 21.5%; all *p* ≤ 0.009). The mean nutritional imbalance score was modestly but significantly higher in the depressive symptoms group (4.9 vs. 4.3; *p* = 0.031). The prevalence of metabolic syndrome did not differ significantly between groups (43.6% vs. 41.8%; *p* = 0.757) (Table 1).

### 3.2. Exploratory Indirect-Effect Analysis

Nutritional imbalance was associated with significantly higher PHQ-9 scores in the total association model (β = 0.216; 95% CI: 0.098, 0.335; *p* < 0.001) and in the direct association model after adjustment for both potential intermediates (β = 0.200; 95% CI: 0.084, 0.316; *p* < 0.001). Oral functional limitation was independently associated with greater PHQ-9 scores (β = 1.278; 95% CI: 0.739, 1.816; *p* < 0.001). Metabolic syndrome score was also associated with PHQ-9 scores (β = 0.156; 95% CI: 0.006, 0.306; *p* = 0.041). Nutritional imbalance was not significantly associated with oral functional limitation (OR = 1.050; 95% CI: 0.974, 1.131; *p* = 0.201) or metabolic syndrome score (β = 0.034; 95% CI: −0.012, 0.080; *p* = 0.151) (Table 2).

Bootstrap-estimated indirect effects via oral functional limitation and metabolic syndrome were directionally consistent but did not reach statistical significance (Table 2).

### 3.3. Joint Exposure Analysis

**Figure 2:**
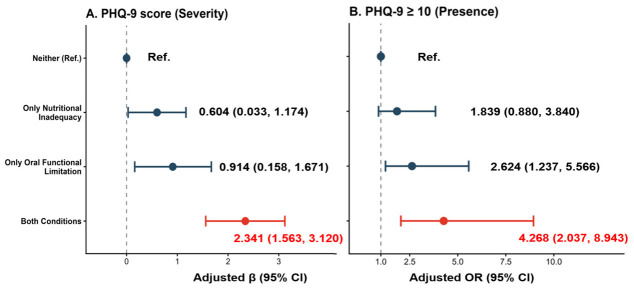
Joint associations of nutritional imbalance and oral functional limitation with depressive symptoms. Adjusted β coefficients are presented for PHQ-9 score (severity), and adjusted odds ratios (ORs) are presented for PHQ-9 ≥ 10 (presence). The reference group consisted of participants with low nutritional imbalance and no oral functional limitation. All models were adjusted for age, sex, education level, household income, marital status, employment status, smoking status, alcohol consumption, aerobic physical activity, and total energy intake. β, regression coefficient; OR, odds ratio; CI, confidence interval; Ref., reference.

Participants with high nutritional imbalance alone had modestly higher PHQ-9 scores than the reference group (β = 0.604; 95% CI: 0.033, 1.174), as did those with oral functional limitation alone (β = 0.914; 95% CI: 0.158, 1.671). Participants with both high nutritional imbalance and oral functional limitation had substantially higher depressive symptom scores (β = 2.341; 95% CI: 1.562, 3.120) and more than four times the odds of PHQ-9 ≥ 10 (OR = 4.268; 95% CI: 2.037, 8.943) compared with those with neither condition. As illustrated in Figure 2, depressive symptom burden increased progressively across joint exposure groups, with the highest burden observed among participants with both high nutritional imbalance and oral functional limitation (Table 3; Figure 2).

In stratified analyses, the association for participants with both conditions was more pronounced in women (OR = 6.148; 95% CI: 2.045, 18.484) than in men (OR = 3.013; 95% CI: 0.865, 10.493), and in those aged <65 years (OR = 5.043; 95% CI: 1.905, 13.348) compared with those aged ≥65 years (OR = 2.376; 95% CI: 0.810, 6.972) (Supplementary Table S2).

Formal tests for multiplicative interaction between high nutritional imbalance and oral functional limitation were not statistically significant for either PHQ-9 score (*p* = 0.109) or clinically significant depressive symptoms (*p* = 0.784) (Supplementary Table S3). Additive interaction analysis showed a positive but non-significant RERI (RERI = 0.805; 95% CI: −1.470, 3.079), with an attributable proportion (AP) of 0.189 and a synergy index (SI) of 1.327, indicating a consistent directional trend across all three metrics, though none reached statistical significance (Supplementary Table S4). These findings are interpreted as joint associations rather than evidence of statistical interaction.

## 4. Discussion

From a nutritional epidemiology perspective, this study demonstrates that nutritional imbalance is independently associated with depressive symptoms among adults living alone, and that co-occurring oral functional limitation was associated with substantially greater depressive symptom burden. Nutritional imbalance, oral functional limitation, and metabolic syndrome score were each independently associated with greater depressive symptom severity. Although the exploratory indirect-effect analyses involving oral functional limitation and metabolic syndrome did not reach statistical significance, the joint exposure analysis showed that participants with both high nutritional imbalance and oral functional limitation experienced the greatest depressive symptom burden. These findings suggest the presence of a vulnerable dual-burden phenotype that may benefit from integrated dietary and oral health screening approaches. Because exposure, potential intermediates, and outcome were measured simultaneously, temporal ordering cannot be confirmed and causal inference is not possible; reverse causation cannot be excluded. For example, depressive symptoms may reduce appetite, dietary motivation, and engagement with oral health behaviors, potentially contributing to both nutritional imbalance and oral functional limitation.

The observed association between nutritional imbalance and depressive symptoms is consistent with previous evidence linking poor dietary quality to adverse mental health outcomes [8,9]. Several biological mechanisms may contribute to this relationship. Micronutrient deficiencies may influence neurotransmitter synthesis, neuroendocrine regulation, inflammatory pathways, and cellular energy metabolism relevant to depression [19,20,21]. In the context of single-person households, nutritional imbalance may additionally reflect behavioral and environmental vulnerabilities associated with eating alone, including lower dietary diversity and greater reliance on energy-dense but nutrient-poor convenience foods [2,3].

The strong independent association between oral functional limitation and depressive symptom severity is also consistent with prior epidemiological findings [12,14]. Oral functional limitation may contribute to depressive symptoms through multiple pathways, including reduced dietary quality secondary to masticatory impairment, communication-related social withdrawal, and poorer health-related quality of life [10,14].

The exploratory indirect-effect analyses did not support formal mediation through either oral functional limitation or metabolic syndrome, as both indirect effects were directionally consistent but did not reach statistical significance. Rather than operating through a single mediated pathway, nutritional imbalance, oral functional limitation, and metabolic risk may function as parallel biological and behavioral stressors that independently contribute to depressive symptom burden. In this sample of adults living alone with a relatively young weighted mean age, subclinical metabolic dysregulation may not yet translate into clinically observable psychiatric outcomes to the extent reported in older populations [15]. The absence of a significant association between nutritional imbalance and oral functional limitation further suggests that oral functional decline in working-age adults living alone may be influenced more strongly by dental care access and oral health behaviors than by nutritional status alone.

Despite the absence of statistically significant multiplicative interaction, the joint exposure analysis identified a clinically meaningful pattern. Participants with both high nutritional imbalance and oral functional limitation had more than fourfold higher odds of clinical depression compared with those with neither condition. This finding suggests a dual-burden vulnerability extending beyond isolated nutritional or oral health risk. When oral functional barriers limit regular consumption of nutrient-rich foods, underlying nutritional inadequacies may be further exacerbated, potentially increasing both physiological and psychosocial vulnerability. The association appeared stronger in women and adults aged <65 years, consistent with patterns reported in previous Korean mental health studies [11,12]. Regarding the clinical significance of the observed associations, while the per-unit association between nutritional imbalance and PHQ-9 score was modest (β = 0.216 per additional nutrient below the EAR), the association observed at the group level was substantially larger. Consistent with the joint exposure findings, participants with both high nutritional imbalance and oral functional limitation had more than fourfold higher odds of clinically significant depressive symptoms (OR = 4.268; 95% CI: 2.037–8.943) compared with those with neither condition. Furthermore, across the observed range of the nutritional imbalance score (0–8), the cumulative difference corresponds to an approximately 1.7-point higher PHQ-9 score. These findings suggest that the accumulation of nutritional deficits and coexisting oral functional limitation may have meaningful implications for depressive symptom burden at the population level, particularly among adults living alone.

Several limitations should be acknowledged. First, the cross-sectional design precludes causal inference, and reverse causation cannot be excluded; for example, depressive symptoms may reduce appetite and dietary motivation, thereby contributing to nutritional imbalance. Second, dietary intake was assessed using a single 24 h dietary recall, which may not adequately represent habitual dietary patterns and is subject to within-person variability and recall bias. Third, the nutritional imbalance index assigns equal weight to each nutrient below the EAR and does not capture the magnitude of individual nutrient shortfalls; a severity-adjusted or weighted index might yield different estimates. Fourth, the indirect-effect analyses were exploratory and relied on assumptions regarding temporal ordering and unmeasured confounding that cannot be fully verified in a cross-sectional design. Fifth, speaking difficulty may be less directly related to nutritional inadequacy than chewing difficulty, and pooling these items may have attenuated the specificity of the oral functional limitation construct. Sixth, approximately 20% of otherwise eligible participants were excluded due to missing data; excluded participants were older and showed higher nutritional imbalance, greater prevalence of oral functional limitation and metabolic syndrome, and lower total energy intake compared with included participants (Supplementary Table S6), suggesting that complete-case analysis may have introduced selection bias. Finally, exclusion of the KNHANES 2023 cycle due to unavailable PHQ-9 data may have further limited sample representativeness.

Despite these limitations, this study has several notable strengths. It used a large nationally representative sample with complex survey design adjustments and validated nutritional and psychiatric measures. The operationalization of oral functional limitation incorporated both masticatory and communicative difficulties, providing a broader assessment than single-item oral health indicators. In addition, the joint exposure framework generated clinically interpretable estimates for a nutritionally and orally vulnerable subgroup. These findings are relevant to public health nutrition and suggest that nutritional support strategies for adults living alone may benefit from integration with oral health assessment and metabolic risk monitoring.

## 5. Conclusions

In conclusion, nutritional imbalance and oral functional limitation were independently and jointly associated with depressive symptom severity among adults living alone in Korea. These findings suggest that the combined burden of dietary insufficiency and oral functional barriers represents an important public health concern. Integrated approaches targeting both dietary quality and oral health may help reduce depressive symptom burden in this nutritionally vulnerable population.

## Figures and Tables

**Table 1:** Characteristics of single-person households according to depressive symptom status.

Variable	Total	No Depressive Symptoms	Depressive Symptoms	*p*-Value
		(PHQ-9 < 10)	(PHQ-9 ≥ 10)	
	*n* = 1572	*n* = 1468	*n* = 104	
**Age, years**	50.5 (19.4)	50.7 (19.5)	48.1 (18.6)	0.232
**Age group**				0.374
19–29	238 (20.8%)	220 (20.6%)	18 (24.2%)	
30–49	283 (26.0%)	262 (25.8%)	21 (29.4%)	
50–64	362 (22.7%)	332 (22.5%)	30 (24.9%)	
≥65	689 (30.5%)	654 (31.2%)	35 (21.5%)	
**Sex**				>0.900
Men	632 (51.0%)	589 (51.0%)	43 (51.5%)	
Women	940 (49.0%)	879 (49.0%)	61 (48.5%)	
**Education level**				>0.900
Elementary or lower	497 (22.0%)	464 (22.1%)	33 (21.3%)	
Middle school	173 (9.2%)	160 (9.1%)	13 (10.4%)	
High school	428 (29.9%)	399 (29.9%)	29 (30.8%)	
College or higher	474 (38.9%)	445 (39.0%)	29 (37.6%)	
**Household income**				<0.001
Lowest	585 (37.2%)	523 (35.3%)	62 (62.8%)	
Lower-middle	386 (23.1%)	367 (23.6%)	19 (16.4%)	
Middle	277 (17.3%)	267 (17.9%)	10 (9.3%)	
Upper-middle	179 (11.9%)	171 (12.1%)	8 (8.7%)	
Highest	145 (10.5%)	140 (11.1%)	5 (2.8%)	
**Marital status**				0.213
Married/partnered	1016 (52.1%)	954 (52.6%)	62 (45.3%)	
Single/separated/widowed	556 (47.9%)	514 (47.4%)	42 (54.7%)	
**Employment status**				0.12
Employed	920 (64.8%)	873 (65.4%)	47 (56.7%)	
Not employed	652 (35.2%)	595 (34.6%)	57 (43.3%)	
**Smoking status**				0.009
Non/former smoker	1262 (76.3%)	1193 (77.2%)	69 (64.3%)	
Current smoker	310 (23.7%)	275 (22.8%)	35 (35.7%)	
**Alcohol consumption**				>0.900
Non-drinker	793 (45.2%)	738 (45.0%)	55 (47.5%)	
Infrequent drinker	651 (45.4%)	612 (45.6%)	39 (43.4%)	
Drinker	128 (9.4%)	118 (9.4%)	10 (9.1%)	
**Aerobic physical activity**				0.060
Did not meet guidelines	859 (48.0%)	810 (48.7%)	49 (38.0%)	
Met guidelines	713 (52.0%)	658 (51.3%)	55 (62.0%)	
**Total energy intake, kcal/day**	1879.7 (911.2)	1881.4 (894.9)	1856.9 (1118.3)	0.850
**Nutritional imbalance score (0–8)**	4.3 (2.4)	4.3 (2.4)	4.9 (2.5)	0.031
**Oral functional limitation**				<0.001
No	1156 (77.4%)	1100 (78.5%)	56 (61.7%)	
Yes	416 (22.6%)	368 (21.5%)	48 (38.3%)	
**Metabolic syndrome**				0.757
No	837 (58.1%)	786 (58.2%)	51 (56.4%)	
Yes	735 (41.9%)	682 (41.8%)	53 (43.6%)	
**PHQ-9 score**	2.8 (4.0)	2.0 (2.3)	14.3 (4.1)	<0.001

Values are weighted mean (SD) for continuous variables and unweighted *n* (weighted %) for categorical variables. The final analytic sample comprised 1572 single-person households (KNHANES 2022: *n* = 687; KNHANES 2024: *n* = 885). KNHANES 2023 was excluded because PHQ-9 was not administered in that cycle. *p*-values were calculated using survey-weighted *t*-test for continuous variables and Rao–Scott chi-square test for categorical variables. PHQ-9, Patient Health Questionnaire-9.

**Table 2:** Exploratory indirect-effect analysis of nutritional imbalance, oral functional limitation, metabolic syndrome, and depressive symptoms.

Association	Metric	Estimate	95% CI	*p*-Value
Nutritional imbalance → Oral functional limitation	OR	1.050	(0.974, 1.131)	0.201
Nutritional imbalance → Metabolic syndrome score	β	0.034	(−0.012, 0.080)	0.151
Oral functional limitation → PHQ-9 score	β	1.278	(0.739, 1.816)	<0.001
Metabolic syndrome score → PHQ-9 score	β	0.156	(0.006, 0.306)	0.041
Total association	β	0.216	(0.098, 0.335)	<0.001
Direct association	β	0.200	(0.084, 0.316)	<0.001
Indirect effect (oral functional limitation)	β	0.058	(−0.043, 0.192)	— ^†^
Indirect effect (metabolic syndrome)	β	0.005	(−0.002, 0.016)	— ^†^

OR, odds ratio; β, regression coefficient; CI, confidence interval; PHQ-9, Patient Health Questionnaire-9. All models were adjusted for age, sex, education level, household income, marital status, employment status, smoking status, alcohol consumption, aerobic physical activity, and total energy intake. ^†^ Bootstrap 95% confidence intervals are reported for indirect effects.

**Table 3:** Joint associations of nutritional imbalance and oral functional limitation with depressive symptoms.

Joint Exposure Group	PHQ-9 Score	PHQ-9 ≥ 10
	β (95% CI)	OR (95% CI)
Low nutritional imbalance + No oral limitation	Ref	Ref
High nutritional imbalance only	0.604 (0.033, 1.174)	1.839 (0.880, 3.840)
Oral limitation only	0.914 (0.158, 1.671)	2.624 (1.237, 5.566)
Both high nutritional imbalance and oral limitation	2.341 (1.562, 3.120)	4.268 (2.037, 8.943)

OR, odds ratio; CI, confidence interval; PHQ-9, Patient Health Questionnaire-9. All models were adjusted for age, sex, education level, household income, marital status, employment status, smoking status, alcohol consumption, aerobic physical activity, and total energy intake.

## Data Availability

Publicly available datasets were analyzed in this study. These data can be found at the Korea Disease Control and Prevention Agency (KDCA) website (https://knhanes.kdca.go.kr/; accessed on 20 May 2026).

## Supplementary Materials

The following supporting information can be downloaded at: https://www.mdpi.com/article/10.3390/nu18132055/s1, Supplementary Table S1, Sensitivity analysis using an alternative PHQ-9 cutoff for depressive symptoms; Supplementary Table S2, Stratified associations of joint nutritional imbalance and oral functional limitation with depressive symptoms; Supplementary Table S3, Multiplicative interaction analysis between high nutritional imbalance and oral functional limitation for depressive symptoms; Supplementary Table S4, Additive interaction analysis between high nutritional imbalance and oral functional limitation for depressive symptoms. Supplementary Table S5. Tertile-based sensitivity analysis of the association between nutritional imbalance and depressive symptoms; Supplementary Table S6. Comparison of baseline characteristics between included and excluded participants.

## Abbreviations

KNHANES	Korea National Health and Nutrition Examination Survey
PHQ-9	Patient Health Questionnaire-9
EAR	Estimated Average Requirement
CI	Confidence Interval
OR	Odds Ratio
