# Supplementary material for: Nutritional Imbalance and Oral Functional Limitation Jointly Associated with Depressive Symptoms in Adults Living Alone: A Nationally Representative Cross-Sectional Study
Source: Nutrients. 2026 Jun 24;18(13):2055. doi: 10.3390/nu18132055 (PMC13363396; doi:10.3390/nu18132055)
Supplement: Supplementary file 1 [file nutrients-18-02055-s001.zip › nutrients-4367580-supplementary.pdf]

Supplementary Table S1. Sensitivity analysis using an alternative PHQ-9 cutoff for depressive symptoms.

Joint exposure group	OR (95% CI)	p-value
Low nutritional imbalance + No oral limitation	Ref.	-
High nutritional imbalance only	1.569 (1.049, 2.345)	0.028
Oral limitation only	1.564 (0.959, 2.549)	0.073
Both high nutritional imbalance and oral limitation	3.554 (2.115, 5.971)	<0.001

Depressive symptoms were defined as a PHQ-9 score of 5 or higher. The reference group was participants with low nutritional imbalance and no oral functional limitation. Models were adjusted for age, sex, education level, household income, marital status, employment status, smoking status, alcohol consumption, aerobic physical activity, and total energy intake.

Supplementary Table S2. Stratified associations of joint nutritional imbalance and oral functional limitation with depressive symptoms.

Subgroup	OR (95% CI)	p-value
Sex		
Men	3.013 (0.865, 10.493)	0.083
Women	6.148 (2.045, 18.484)	0.001
Age group		
<65 years	5.043 (1.905, 13.348)	0.001
≥65 years	2.376 (0.810, 6.972)	0.114

The table presents adjusted odds ratios for depressive symptoms among participants with both high nutritional imbalance and oral functional limitation, compared with participants with low nutritional imbalance and no oral functional limitation. Depressive symptoms were defined as a PHQ-9 score of 10 or higher. Models were adjusted for age, education level, household income, marital status, employment status, smoking status, alcohol consumption, aerobic physical activity, and total energy intake. In sex-stratified analyses, sex was not included as a covariate. In age-stratified analyses, age was retained as a continuous covariate.

Supplementary Table S3. Multiplicative interaction analysis between high nutritional imbalance and oral functional limitation for depressive symptoms.

Outcome	Estimate	95% CI	p-value
PHQ-9 score	β = 0.823	(−0.185, 1.830)	0.109
PHQ-9 ≥10	OR = 0.885	(0.366, 2.136)	0.784

Models were adjusted for age, sex, education level, household income, marital status, employment status, smoking status, alcohol consumption, aerobic physical activity, and total energy intake. Multiplicative interaction was evaluated using a product term between high nutritional imbalance and oral functional limitation in survey-weighted regression models. For PHQ-9 ≥10, odds ratios were derived by exponentiating logistic regression coefficients. CI, confidence interval; OR, odds ratio; PHQ-9, Patient Health Questionnaire-9

Supplementary Table S4. Additive interaction analysis between high nutritional imbalance and oral functional limitation for depressive symptoms.

Measure	Estimate	95% CI
RERI	0.805	(−1.470, 3.079)
AP	0.189	-
SI	1.327	-

RERI, relative excess risk due to interaction; AP, attributable proportion due to interaction; SI, synergy index. RERI = $OR_{11} - OR_{10} - OR_{01} + 1$, where OR_{11} denotes the odds ratio for both high nutritional imbalance and oral functional limitation, OR_{10} for high nutritional imbalance only, and OR_{01} for oral functional limitation only. AP = RERI / OR_{11}. SI = $(OR_{11} - 1) / [(OR_{10} - 1) + (OR_{01} - 1)]$. The 95% confidence interval for RERI was estimated using the delta method. AP and SI confidence intervals were not estimated. Values greater than zero (RERI, AP) or greater than one (SI) indicate positive additive interaction. The reference group was participants with low nutritional imbalance and no oral functional limitation. Models were adjusted for age, sex, education level, household income, marital status, employment status, smoking status, alcohol consumption, aerobic physical activity, and total energy intake.

Supplementary Table S5. Tertile-based sensitivity analysis of the association between nutritional imbalance and depressive symptoms.

Outcome	Exposure	Estimate	95% CI	p-value
PHQ-9 score	Second tertile vs. first tertile	0.555	(0.011, 1.099)	0.046
PHQ-9 score	Third tertile vs. first tertile	1.326	(0.600, 2.053)	<0.001
Clinically significant depressive symptoms (PHQ-9 ≥10)	Second tertile vs. first tertile	1.850	(0.917, 3.730)	0.085
Clinically significant depressive symptoms (PHQ-9 ≥10)	Third tertile vs. first tertile	2.854	(1.347, 6.044)	0.006

Abbreviations: CI, confidence interval; OR, odds ratio; PHQ-9, Patient Health Questionnaire-9. Estimates represent β coefficients for PHQ-9 score and odds ratios (ORs) for clinically significant depressive symptoms (PHQ-9 ≥10). Models were adjusted for age, sex, education level, household income, smoking status, alcohol consumption, aerobic physical activity, and total energy intake. The first tertile (T1) was used as the reference group.

Supplementary Table S6. Comparison of baseline characteristics between included and excluded participants.

Variable	Included (N = 1,572)	Excluded (N = 391)	p-value
**Age, years**	56.3 (19.1)	64.3 (16.7)	< 0.001
**Sex**			< 0.001
Men	632 (40.2%)	114 (29.2%)	
Women	940 (59.8%)	277 (70.8%)	
**Education level**			0.007
Elementary or lower	497 (31.6%)	103 (40.1%)	
Middle school	173 (11.0%)	16 (6.2%)	
High school	428 (27.2%)	74 (28.8%)	
College or higher	474 (30.2%)	64 (24.9%)	
**Household income**			0.400
Lowest	585 (37.2%)	140 (38.1%)	
Lower-middle	386 (24.6%)	104 (28.3%)	
Middle	277 (17.6%)	60 (16.3%)	
Upper-middle	179 (11.4%)	37 (10.1%)	
Highest	145 (9.2%)	26 (7.1%)	
**Smoking status**			0.300
Non/former smoker	1,262 (80.3%)	247 (82.9%)	
Current smoker	310 (19.7%)	51 (17.1%)	
**Alcohol consumption**			< 0.001
Non-drinker	793 (50.4%)	269 (68.8%)	
Infrequent drinker	651 (41.4%)	102 (26.1%)	
Drinker	128 (8.1%)	20 (5.1%)	
**Aerobic physical activity**	713 (45.4%)	61 (33.3%)	0.002
**Total energy intake, kcal/day**	1,758.6 (855.1)	1,498.9 (718.3)	< 0.001
**Nutritional imbalance score (0–8)**			< 0.001
0	87 (5.5%)	13 (4.3%)	
1	116 (7.4%)	14 (4.7%)	
2	190 (12.1%)	24 (8.0%)	
3	192 (12.2%)	27 (9.0%)	
4	201 (12.8%)	31 (10.4%)	
5	200 (12.7%)	27 (9.0%)	
6	165 (10.5%)	40 (13.4%)	
7	161 (10.2%)	34 (11.4%)	
8	260 (16.5%)	89 (29.8%)	
**Oral functional limitation, n (%)**	416 (26.5%)	96 (32.3%)	0.038
**Metabolic syndrome, n (%)**	735 (46.8%)	130 (55.6%)	0.012
**PHQ-9 score**	2.8 (4.1)	3.5 (5.1)	0.200

Abbreviations: PHQ-9, Patient Health Questionnaire-9. Values are presented as mean (SD) for continuous variables and n (%) for categorical variables. P-values were calculated using the Wilcoxon rank-sum test for continuous variables and Pearson's chi-squared test for categorical variables.
